# Serological Investigation and Genetic Characteristics of Pseudorabies Virus in Hunan Province of China From 2016 to 2020

**DOI:** 10.3389/fvets.2021.762326

**Published:** 2021-12-16

**Authors:** Yuan Lin, Lei Tan, Changjian Wang, Shicheng He, Ling Fang, Zicheng Wang, Yating Zhong, Kun Zhang, Daoxin Liu, Qing Yang, Aibing Wang

**Affiliations:** ^1^Hunan Provincial Key Laboratory of Protein Engineering in Animal Vaccines, College of Veterinary Medicine, Hunan Agricultural University, Changsha, China; ^2^Hunan Provincial Center for Animal Disease Control and Prevention, Changsha, China; ^3^School of Public Administration and Law, Hunan Agricultural University, Changsha, China; ^4^PCB Biotechnology LLC, Rockville, MD, United States

**Keywords:** pseudorabies virus, serological investigation, viral isolation, phylogenetic analysis, Hunan Province

## Abstract

Pseudorabies (PR), caused by variant pseudorabies virus (PRV), is an economically important viral disease in China. Recently, PRV infection in humans has also received attention worldwide. To investigate the PRV infection in Hunan province, China, we collected a total of 18,138 serum specimens from 808 PRV-vaccinated pig farms cross this region during 2016–2020, and we detected the presence of PRV glycoprotein B (gB) and gE-specific antibodies. The enzyme-linked immunosorbent assay (ELISA) results revealed that 80.47% (14,596/18,138, 95 CI 79.9–81.0) and 23.55% (4,271/18,138, 95 CI 22.9–24.2) of serum samples were positive for PRV gB and gE-specific antibodies, respectively. Further analysis indicated that the seroprevalence of wild PRV infection was associated with the season and breeding scale (*p* < 0.01). In addition, five PRV strains were isolated from PRV-positive samples in Vero cells and the virus titers varied from 10^6.5^ to 10^7.51^ TCID_50_/0.1 ml. The phylogenetic analysis revealed that one isolate was a classical strain of PRV genotype II, and four other isolates belonged to the variants of genotype II. Collectively, the data indicate that the prevalence of PRV remains high in pigs in Hunan province, and the variant PRV strains are the major genotypes affecting the development of the pig industry.

## Introduction

Pseudorabies virus (PRV) is a double-stranded linear DNA virus with ~143 kb and encoding more than 70 proteins belonging to the genus *Varicellovirus* of the subfamily *Alphaherpesviridae* (family *Herpesviridae*) (1). Pseudorabies (PR) or Aujeszky's disease caused by PRV is a major threat to the pig industry in China, and the symptoms of PR are mainly characterized by reproductive failure in sows, fatal encephalitis and neurological symptoms in newborn piglets, and respiratory disorders in fattening pigs (2, 3).

Pig is the natural host and reservoir for PRV, while this pathogen can also infect various mammals, such as ruminants (4, 5), carnivores (6), bears (7), etc. Recently, a PRV strain has been isolated from an acute human encephalitis case, suggesting that humans may be potential PRV hosts (8).

With the wide application of glycoprotein E (*gE*)-deleted PRV vaccines, PR had largely been controlled worldwide and even eradicated in Mexico, Canada, and New Zealand (1, 9). However, PRV-triggering diseases have frequently been documented in Bartha-K61-immunized swine populations in China since late 2011 (10, 11). Molecular analysis revealed that the causative agents were identified as PRV variants, which have high genetic variations in some antigenic regions (as *gC, gD*, and *gE*) compared to the classic PRV strains (12, 13).

PRV infection causes significant morbidity and mortality in swine and huge economic losses to the pig industry. It is also a potential threat to public health, since it can be transmitted from pigs to other animal species, even humans (8). Thus, investigation of the PRV prevalence and its genetic variations is beneficial to control this disease. Although the prevalence of PRV has been reported in several provinces or regions of China (14–17), data on PRV epidemiology is unavailable in Hunan province, which is located in Middle-South China.

To address this issue, serum samples were collected from 2016 to 2020 in Hunan province, and the epidemiology of PRV was investigated. Meanwhile, five new PRV strains were identified and their genetic characteristics were analyzed.

## Materials and Methods

### Specimen Collection

**Figure 1:**
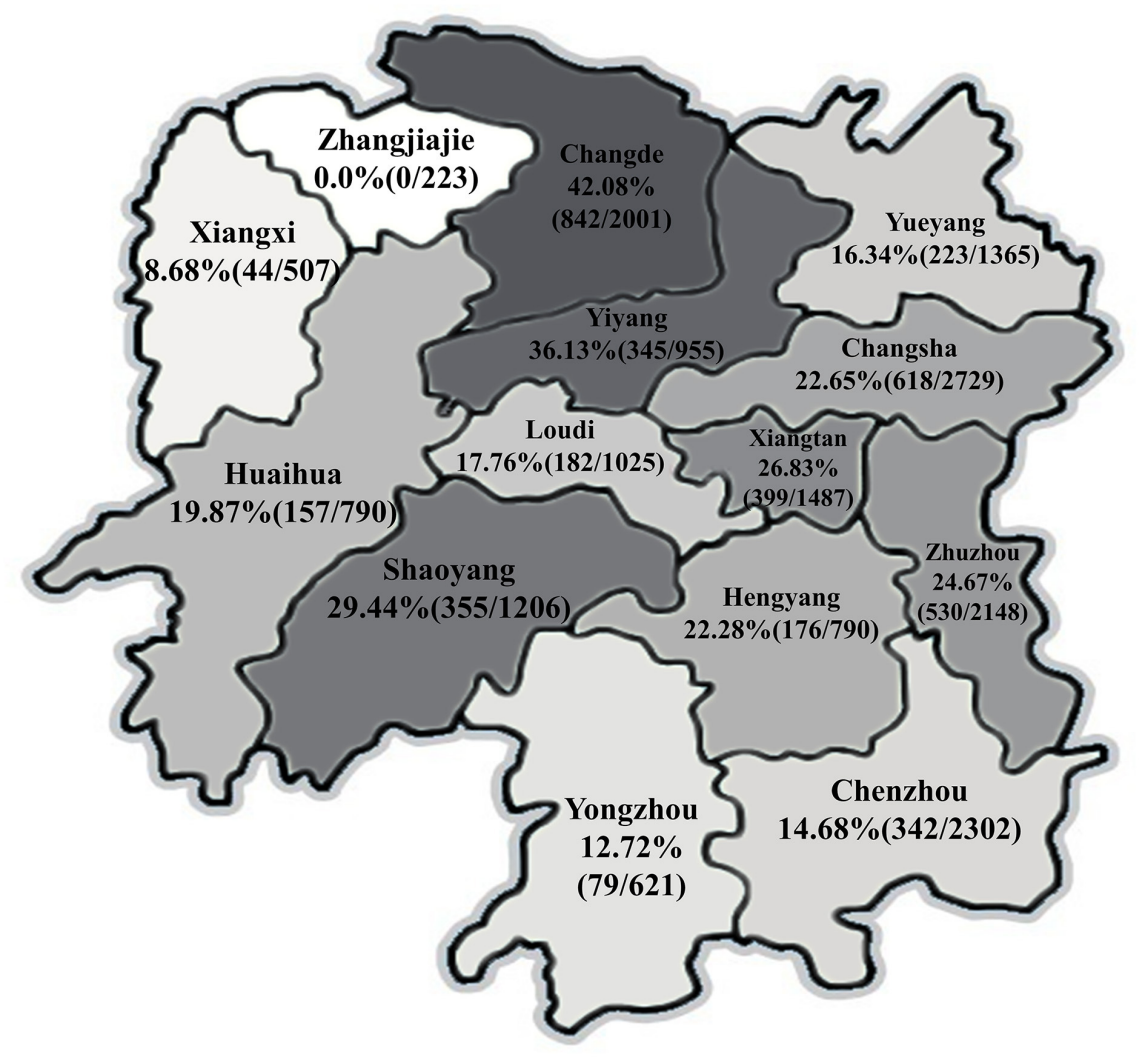
Seroprevalence of pseudorabies virus (PRV) in different geographical regions in Hunan province of China.

A total of 18,138 serum samples were collected from piglets, nursery pig, fatting pig, sow, gilts, and boars in 808 PRV-vaccinated pig farms cross the Hunan province from 2016 to 2020 (Figure 1). According to the breeding scale, 10, 30, and 60~90 samples were collected from each small (<500 pigs), medium (500~2,000 pigs), and large-scaled farm (>2,000 pigs), respectively. Additionally, lymph node and brain tissue samples were collected from 42 piglets with clinical signs of diarrhea, vomiting, and encephalitis in 25 farms, and the presence of PRV nucleic acids was detected. The detailed information of each sample including the herd location, collection date, and breeding scale were recorded.

### Serological Detection

Anti-gE antibody levels in serum were determined by using commercial blocking ELISA Kits (Cat: CP144, IDEXX Laboratories, Westbrook, ME) according to the manufacturer's instructions, which can differentiate the vaccine strain from the wild PRV strains. The presence of anti-gB antibodies was also detected using commercial IDEXX blocking ELISA Kits (Cat: EP327).

### Virus Detection

Viral DNA and RNA were extracted from the tissue samples using commercial kits (Takara, Dalian, China) according to the manufacturer's instructions, and cDNAs were synthesized using a RevertAid First Strand cDNA Synthesis Kit (Thermo Fisher Scientific, USA). The presences of PRV, porcine circovirus type 2 (PCV2), PCV3, classical swine fever virus (CSFV), porcine reproductive and respiratory syndrome virus (PRRSV), and porcine epidemic diarrhea virus (PEDV) were detected by polymerase chain reaction (PCR) or reverse-transcription (RT)-PCR as described previously (18). Specific primers were shown in Supplementary Table S1.

### Virus Isolation and Identification

The homogenates of PRV-positive mixed lymph node and brain tissues were subjected to three freezing-thawing cycles. The supernatants were harvested after centrifugation and passed through 0.22 μm filters (EMD Millipore, Billerica, MA, USA). Monolayers of African green monkey kidney (Vero) cells were cultured with the supernatants for 1.5 h followed by maintenance in DMEM (Gibco, USA) supplemented with 10% fetal bovine serum (Gibco), 100 μg/ml streptomycin, and 100 IU/mL penicillin at 37°C in a 5% CO_2_ incubator. The cultures containing the viruses were freeze-thawed three times when cytopathic effects (CPE) were observed in 80% of the cells. The viruses were further purified by plaque assays and confirmed by PCR assay. Viral titers (50% tissue culture infectious doses per mL, TCID_50_) were titrated by Reed-Muench method in Vero cells.

### Sequencing and Phylogenetic Analysis

Viral DNAs were extracted from the Vero cells infected with the purified isolates, and the full-lengths of *gE, gC*, and *TK* genes were amplified by PCR as described previously (11). The specific primers were listed in Supplementary Table S1. Fragments were sequenced and submitted to the GenBank database (Supplementary Table S2). The nucleotide sequences and the corresponding amino acid variations of these genes were analyzed using DNAStar version 7.10 (Lasergene DNAStar software). Phylogenetic trees based on the *gE, TK*, and *gC* genes were reconstructed using the maximum likelihood (ML) method in MEGA 7.0 software [time-reversible (GTR) model; 1,000 bootstrap replicates]. The PRV reference strains retrieved from NCBI database are listed in Supplementary Table S2.

### Statistical Analysis

The associations between the sero-prevalence of PRV-gE and gB antibodies and factors, including season, breeding scale, and pig herd group, were analyzed using Chi-square test in SPSS 20.0 software (IBM, Chicago, IL, USA). The 95% confidence intervals (CIs) were investigated. Meanwhile, *p*-values < 0.05 were determined as statistical significance.

## Results

### Seroprevalence of PRV in Hunan Province

In the present study, the investigated pig farms performed routine immunization with either attenuated or inactivated PRV Bartha-K61 strain vaccine, or attenuated or inactivated PRV HB-98 strain vaccine. Data obtained from the ELISA assay demonstrated that 68.56% (554/808, 95% CI 65.4–71.8) of the investigated pig farms meet the national requirement, in which the PRV-gB sero-positive rate of the pig population should be higher than 70% after immunization, while the proportion of PRV-gE-positive farms was up to 43.19% (349/808, 95% CI 39.8–46.6), indicating that the current vaccines could not provide complete protection. The seroprevalence rate of PRV-gB was 80.47% (14,596/18,138, 95% CI 79.9–81.0) (Table 1). Moreover, the seroprevalence of PRV-gE kept at a high level, and the average rate was 23.55% (4,271/18,138, 95% CI 22.9–24.2), with a gradual increased trend throughout the investigation period (Table 1).

**Table 1:** Factors associated with the seroprevalence of PRV-gB and PRV-gE in pigs in Hunan province, China.

**Factor**	**Category**	**No. sample**	**gB antibody**			**gE antibody**		
			**No. positive**	**% (95% CI)**	***P*-value**	**No. positive**	**% (95% CI)**	***P*-value**
Year	2016	3,651	2,653	72.67 (71.2-74.1)	Reference	727	19.91 (18.6-21.2)	Reference
	2017	1,821	1,594	87.53 (86.0-89.1)	<0.01	432	23.72 (21.8-25.7)	0.01
	2018	4,202	3,206	76.30 (75.0-77.6)	<0.01	982	23.37 (22.1-24.6)	<0.01
	2019	4127	3,462	83.89 (92.8-85.0)	<0.01	1,026	24.86 (23.5-26.2)	<0.01
	2020	4,337	3,681	84.87 (83.8-85.9)	<0.01	1,104	25.46 (24.2-26.8)	<0.01
Pig herd	Piglets	1,059	898	84.80 (82.6-87.0)	<0.01	250	23.61 (21.0-26.2)	<0.01
	Nursery pigs	2,015	1,686	83.67 (82.1-85.3)	<0.01	535	26.55 (24.6-28.5)	<0.01
	Fattening pigs	5,419	3,260	60.16 (58.9-61.5)	Reference	1,010	18.64 (17.6-19.7)	Reference
	Sows	5,921	5,505	92.97 (92.3-93.6)	0.01	1,650	27.87 (26.7-29.0)	<0.01
	Gilts	2,370	1,959	82.66 (81.1-84.2)	<0.01	474	20.00 (18.4-21.6)	0.159
	Boars	1,354	1,288	95.13 (94.0-96.3)	<0.01	352	26.00 (23.7-28.3)	<0.01
Season	Spring	7,476	5,763	77.09 (76.1-78.0)	Reference	1,732	23.17 (22.2-24.1)	<0.01
	Summer	2,490	2,080	83.53 (82.1-85.0)	<0.01	555	22.29 (20.7-23.9)	<0.01
	Autumn	4,669	3,937	84.32 (83.3-85.4)	<0.01	1,328	28.44 (27.1-29.7)	<0.01
	Winter	3,503	2,816	80.39 (79.1-81.7)	<0.01	656	18.72 (17.4-20.0)	Reference
Breeding scale	Large	10,434	9,165	87.84 (87.2-88.5)	<0.01	2,968	28.45 (27.6-29.3)	<0.01
	Medium	4,982	3,876	77.80 (76.6-79.0)	<0.01	769	15.44 (14.4-16.4)	Reference
	Small	2,722	1,555	57.13 (55.3-59.0)	Reference	534	19.62 (18.1-21.1)	<0.01
Total		18,138	14,596	80.47 (79.9-81.0)		4,271	23.55 (22.9-24.2)	

As shown in Figure 1, the average seroprevalence rate of PRV-gE displayed a pronounced regional variation in Hunan province, no PRV-gE-positive sample was detected in the region of Zhangjiajie, which is located in the northwest of Hunan, while the positive rates exceeded 10% in 12 of the 14 regions, and more than 42% of the investigated pigs were PRV-gE positive in Changde.

Seasonal variation and other factors with a potential association with PRV were evaluated. Seasonal variations in PRV infection rate were evident and peaked in autumn with a PRV-gE positive rate of 28.44% (1,328/4,669, 95% CI 27.1–29.7), while the lowest seroprevalence was in winter (18.73%, 656/3,503, 95% CI 17.4–20.0) (Table 1). In term of breeding scale, the infection rates in large-scale (28.45%, 2,968/10,434, 95% CI 27.6–29.3) and small farms (19.62%, 534/2,722, 95% CI 18.1–21.1) were significantly higher than that in medium-sized farms (15.44%, 769/4,982, 95% CI 14.4–16.4) (*p* < 0.01) (Table 1). In different pig herds, except the fattening pigs (60.16%, 3,260/5,419, 95% CI 58.9–61.5), more than 80% of the investigated piglets, nursery pigs, sows, gilts, and boars were PRV-gB antibody positive. Moreover, the overall PRV-gE seroprevalence in fattening pigs (18.64%, 1,010/5,419, 95% CI 17.6–19.7) was significantly lower than those in piglets (23.61%, 250/1,059, 95% CI 21.0–26.2), nursery pigs (26.55%, 535/2,015, 24.6–28.5), sows (27.87%, 1,650/5,921, 95% CI 26.7–29.0), and boars (26.00%, 352/1,354, 95% CI 26.7–29.0) (*p* < 0.01) (Table 1).

### PRV Isolation

The detection of PRV nucleic acids demonstrated that 7 of the 42 tissue samples were PRV-gE positive and negative for the other detected pathogens (Supplementary Figure S1). To further investigate the genome characteristics of PRV strains prevalent in Hunan province, five PRV strains were successfully isolated and confirmed by PCR. The titers in Vero cells varied from 10^6.50^ to 10^7.51^ TCID_50_/0.1 ml. The isolates were designated as HuN-HH/2020, HuN-XT/2020, HuN-XX/2020, HuN-LD/2019, and HuN-YY/2018 according to the collection region and year.

### Phylogenetic Analysis

**Figure 2:**
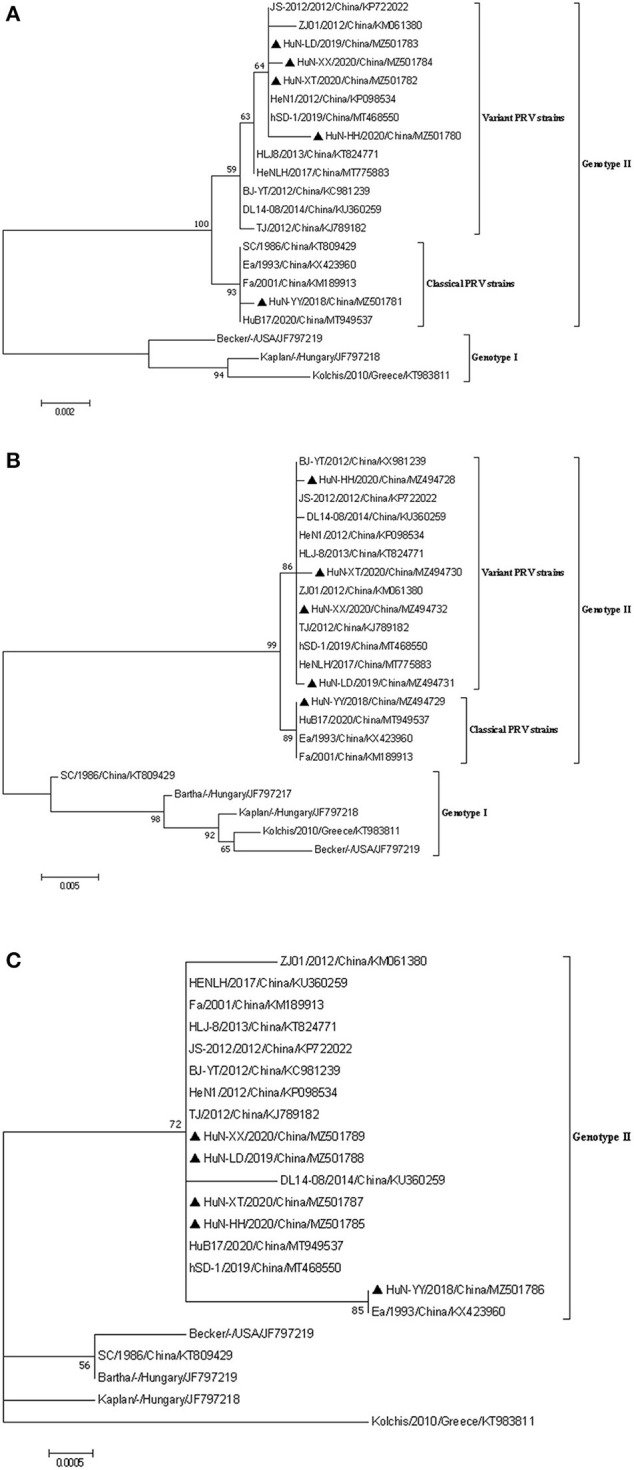
Phylogenetic analysis based on the nucleotide sequences of *gE* **(A)**, *gC* **(B)**, and *TK* **(C)** gene of the five PRV isolates and other reference strains. The phylogenetic trees were generated using the maximum likelihood method [time-reversible (GTR) model; 1,000 bootstrap replicates] in MEGA 7.0 software. The black triangle represented the five PRV isolates.

Phylogenetic analysis based on the PRV *gE, gC*, and *TK* genes revealed that most of referenced PRV strains prevalent in China belonged to the genotype II (Figures 2A,B), and the five strains isolated in Hunan formed a monophyletic clade (genotype II), which was distinct from PRVs of genotype I (Figures 2A–C). Among the five isolates, the HuN-YY/2018 was a classical strain of genotype II, and the other four were grouped as the PRV variants (Figures 2A,B).

### Analysis of PRV *gC, TK*, and *gE*

The full-length sequences of PRV-*gC* (1,464 bp), *gE* (1,737-1,740 bp) and *TK* (963 bp) of the isolates were amplified by PCR, which shared more than 99.5% identity in nucleotide sequence between each other, and the amino acid sequence identities were 99.2~99.8% in gC, 98.4~100.0% in gE, and no variation in TK (Supplementary Table S3). Further sequence analysis showed that the maximum amino acid sequence divergences in *gE, gC*, and *TK* were 1.6, 5.6, and 0.3% when compared with the PRV genotype II strains, respectively (Supplementary Table S3).

In addition, similar to PRV genotype II strains, seven continuous amino acids insertion (^64^AASTPAA^70^) was identified in the gC proteins of the five isolates by amino acid sequence alignment analysis when compared to genotype I strains. Compared with PRV variant strains, the gE protein of the HuN-YY/2018 isolate as the Chinese classical strains had one amino acid deletion at position 498 (D).

## Discussion

PR had largely been controlled in some regions between 2005 to 2010 with the application of gE-deleted vaccine (17). However, this disease was frequently reported in Bartha-K61-vaccinated pig farms in late 2011, causing high mortality in newborn piglets (19). Despite extensive efforts in eradication, PR is still a serious threat to the pig industry in China (17, 20). It has been listed in the “Mid- and Long-term Animal Disease Prevention and Control Program in China” (1).

Recently, we collected data reported from 2011 to 2020 and found that the overall seropositive rate of PRV-gE was 29.87% (76,553/256,326) in China (13). PRV prevalence in pigs varied in different regions in China. In Henan province, 30.14% (1,419/4,708) of samples collected during 2018–2019 were gE-seropositive (17), while the serum gE-positive rate was 16.3% (3,067/18,815) in Heilongjiang province from 2013 to 2018 (21). All data indicate that seroprevalence of variant PRV is still high in China. However, the molecular prevalence of PRV is relatively low in China. Recent research showed that PRV-gE yielded an 8.27% (1,345/16,256) of PRV-positive rate in tissue samples from PRV-suspected pig farms in different regions of China (11). Considering that PRV can be transmitted to other mammal species, the prevalence of PR in pig population needs more attention.

The present results revealed that the PRV-gE seropositive rate had a wide range from 0.0 to 41.28% in different geographical regions of Hunan province, which was also reported in other studies (11, 17). The geographical difference might be associated with sampling size, transportation, breeding scale, and conditions.

Environmental stress is a major threat to animals. The seasonal variation showed that PRV seroprevalence occurred more in autumn than other seasons from 2016 to 2020 in Hunan province. The variable temperature in autumn may partially state this result, and similar results were also reported in a previous study (17). Meanwhile, as reported in Zhou's study (21), the lowest PRV seroprevalence was also observed in fattening pigs, which might be associated with the limited breeding time.

In addition, the PRV-gE seroprevalence rate in large farms was higher than those in medium and small ones. High-density feeding and ASFV prevalence might contribute to this point. High-density feeding increased the difficulty of disease prevention and control, such as vaccine immunization assessment, frequent staff mobility, etc. The sow population decreased a lot owing to the prevalence of ASFV in China (22, 23). Some pig farms mainly focused on ASF prevention and control, and many pigs were introduced into large farms to keep the breeding scale, which increase the risks of PRV introduction. Meanwhile, some medium and small-sized farms were closed due to the environmental issue and the risk of ASF. The data above indicated that there was no significant correlation between vaccination rate and wild virus infection.

PRV binds to the cellular surface via gC, an important target for neutralizing antibody (24). PRV-gE and TK are associated with virulence (25). Sequence alignments showed that except for TK, individual amino acid mutations were found in gC and gE compared with the reference strains. Changes in gC or gE might affect the virulence of the isolates, which needs further investigation.

PRV strains are divided into two genotypes according to their genetic characteristics. Most PRV isolates from China and other countries in Asia are classified into genotype II, while the prevalent PRV strains in Europe and America belong to genotype I (13). The present results showed that PRV classical strains were also prevalent in pig herds in Hunan province. Though Bartha-K61 vaccine can provide complete protection against the classical strain (10, 19), some factors may contribute to vaccine failure, such as co-infection with PRRSV (21), indicating that the PR eradication or control cannot only rely on vaccination. Additionally, PRV recombinant isolates derived from genotype I and genotype II strains or classical and variant strains were often identified (26, 27). Thus the prevalence of classic strains and variant PRVs and wide application of the attenuated vaccine strain Bartha-K61 may increase the possibility of PRV genomic recombination, which needs further investigation.

## Conclusion

In summary, this study investigated the seroprevalence and genetic characteristics of PRV from 2016 to 2020 in Hunan province of China and revealed that both classic and variant PRV strains were circulating in this province and PR was still widely prevalent in recent years, and the eradication of PR could not only depend on vaccination. Therefore, the information herein should facilitate the future evaluation of PRV prevalence in Hunan province. Additionally, efficient measures including new vaccine development and fine breeding management should be taken for the control of PR.

## Data Availability Statement

The datasets presented in this study can be found in online repositories. The names of the repository/repositories and accession number(s) can be found in the article/Supplementary Material.

## Ethics Statement

This study was approved by the Animal Ethics Committee of Hunan Agricultural University, Hunan, China (No. 43321503).

## Author Contributions

AW, QY, LT, and YL: conceptualization and writing—original draft preparation. AW, LT, and YL: data curation, visualization, and validation. LT and YL: formal analysis. LT, YL, CW, SH, LF, ZW, YZ, KZ, and DL: investigation and methodology. AW and QY: funding. All authors have read and agreed to the published version of the final manuscript.

## Funding

This research was supported by National Natural Science Foundation of China (Grant Nos. 31772819 and 31972761), the Hunan Provincial Natural Science Foundation of China (2020JJ4041), and the Postgraduate Scientific Research Innovation Project of Hunan Province (CX20200659).

## Conflict of Interest

AW was employed by company PCB Biotechnology LLC. The remaining authors declare that the research was conducted in the absence of any commercial or financial relationships that could be construed as a potential conflict of interest.

## Publisher's Note

All claims expressed in this article are solely those of the authors and do not necessarily represent those of their affiliated organizations, or those of the publisher, the editors and the reviewers. Any product that may be evaluated in this article, or claim that may be made by its manufacturer, is not guaranteed or endorsed by the publisher.
