# Supplementary material for: Serological Investigation and Genetic Characteristics of Pseudorabies Virus in Hunan Province of China From 2016 to 2020
Source: Front Vet Sci. 2021 Dec 16;8:762326. doi: 10.3389/fvets.2021.762326 (PMC8716618; doi:10.3389/fvets.2021.762326)
Supplement: Supplementary file 1 [file Table_1.DOC]

**Supplementary Table 1** Primers used for PCR amplification in this study

| **Primer** | **Sequence 5’-3’** | **Binding position** | **Length** | **Purpose** | **Reference sequence** |
| --- | --- | --- | --- | --- | --- |
| PRV-detection-F | CGACTACGCCGACTACTACG | 119961-120406 | 446 | Detection of PRV | [MK806387](https://www.ncbi.nlm.nih.gov/nucleotide/MK806387.1?report=genbank&log$=nuclalign&blast_rank=1&RID=ZU03AUKV016) |
| PRV-detection-R | CGAAGATGTCAGAGGGCGAG |
| PCV2-detection-F | CCATATGAAATAAATTACTGAG | 975-1737 | 763 | Detection of PCV2 | AY321991 |
| PCV2-detection-R | CAGCGCACTTCTTTCGTTTTGAG |
| PCV3-detection-F | TTACTTAGAGAACGGACTTGTAACG | 1339-1989 | 649 | Detection of PCV3 | MN075133 |
| PCV3-detection-R | AAATGAGACACAGAGCTATATTCAG |
| CSFV-detection-F | AACATGGATGGTGTAACTGG | 1408-1728 | 321 | Detection of CSFV | AY805221 |
| CSFV-detection-R | TTCTCTATAGTGTTGGTCATTCC |
| PRRSV-detection-F | GAGTTTCAGCGGAACAATGG | 14271-14703 | 433 | Detection of PRRSV | KC527830 |
| PRRSV-detection-R | GCCGTTGACCGTAGTGGAG |
| PRV-TK-F | TCGTAGAAGCGGTTGTGGC | 59458-60591 | 1134 | Sequencing | [MK806387](https://www.ncbi.nlm.nih.gov/nucleotide/MK806387.1?report=genbank&log$=nuclalign&blast_rank=1&RID=ZU03AUKV016) |
| PRV-TK-R | GGCAAACTTTATTGGGATGA |
| PRV-gE-F | TTTGTGGGTGGCGTTTTATCTC | 123287-125244 | 1958 | Sequencing | [MK806387](https://www.ncbi.nlm.nih.gov/nucleotide/MK806387.1?report=genbank&log$=nuclalign&blast_rank=1&RID=ZU03AUKV016) |
| PRV-gE-R | AGCAGTCCGAGTCGTCCTGG |
| PRV-gC-F | CGCCACTAGCATTAAATCC | 53582-55372 | 1791 | Sequencing | KU900059 |
| PRV-gC-R | TCTCGCAGATGATGTCCC |

**Supplementary Table 2** Information of PRV isolates obtained in this study and reference strains

| Strain | Year of isolation | Region of isolation | GenBank accession | Genotype (Note) |
| --- | --- | --- | --- | --- |
| Bartha | - | Hungary | JF797217 (complete genome) | Genotype I |
| Becker | - | USA | JF797219 (complete genome) | Genotype I |
| Kaplan | - | Hungary | JF797218 (complete genome) | Genotype I |
| Kolchis | 2010 | Greece | KT983811 (complete genome) | Genotype I |
| Ea | 1993 | China/Hubei | KX423960 (complete genome) | Genotype II |
| Fa | 2001 | China/Fujian | KM189913 (complete genome) | Genotype II |
| SC | 1986 | China/Sichuan | KT809429 (complete genome) | Genotype II |
| HLJ-8 | 2013 | China/Heilongjiang | KT824771 (complete genome) | Genotype II |
| TJ | 2012 | China/Tianjin | KJ789182 (complete genome) | Genotype II |
| HeN1 | 2012 | China/Henan | KP098534 (complete genome) | Genotype II |
| BJ/YT | 2012 | China/Beijing | KC981239 (complete genome) | Genotype II |
| JS-2012 | 2012 | China/Jiangsu | KP722022 (complete genome) | Genotype II |
| ZJ01 | 2012 | China/Zhejiang | KM061380 (complete genome) | Genotype II |
| HuB17 | 2020 | China/Hubei | MT949537 (complete genome) | Genotype II |
| hSD-1 | 2019 | China | MT468550 (complete genome) | Genotype II |
| HeNLH | 2017 | China/Henan | MT775883 (complete genome) | Genotype II |
| DL14/08 | 2014 | China | KU360259 (complete genome) | Genotype II |
| HuN-HH/2020 | 2020 | China/Hunan | MZ494728 (gC), MZ501780 (gE), MZ501785 (TK) | Genotype II |
| HuN-YY/2018 | 2018 | China/Hunan | MZ494729 (gC), MZ501781 (gE), MZ501786 (TK) | Genotype II |
| HuN-XT/2020 | 2020 | China/Hunan | MZ494730 (gC), MZ501782 (gE), MZ501787 (TK) | Genotype II |
| HuN-LD/2019 | 2019 | China/Hunan | MZ494731(gC), MZ501783 (gE), MZ501788 (TK) | Genotype II |
| HuN-XX/2020 | 2020 | China/Hunan | MZ494732 (gC), MZ501784 (gE), MZ501789 (TK) | Genotype II |

**Supplementary Table 3** Sequence identity in the *gC*, *gE*, and *TK* genes of PRV isolates identified in the present study

| **PRV strains** | **Nucleotide** | | | **Amino acid** | | |
| --- | --- | --- | --- | --- | --- | --- |
|  | ***gC*** | ***gE*** | ***TK*** | ***gC*** | ***gE*** | ***TK*** |
| PRV isolates identified in this study | 99.7~99.9% | 99.5~100.0% | 99.8~100.0% | 99.2~99.8% | 98.4~100.0% | 100.0% |
| Compared with Chinese traditional PRV strains | 99.6~100.0% | 99.5~99.9% | 98.3-99.9% | 94.4~100.0% | 98.4~99.7% | 99.7% |
| Compared with Chinese variant PRV strains | 99.7~99.9% | 99.5~100.0% | 99.5~100.0% | 99.2~99.6% | 98.4~100.0% | 99.7~100.0% |
| Compared with European and American PRV strains | 95.8~96.2% | 97.7~98.0% | 42.7~43.1% | 92.5~93.1% | 95.2~95.8% | 99.1~99.4% |
