# Supplementary material for: Serological Investigation and Genetic Characteristics of Pseudorabies Virus in Hunan Province of China From 2016 to 2020
Source: Front Vet Sci. 2021 Dec 16;8:762326. doi: 10.3389/fvets.2021.762326 (PMC8716618; doi:10.3389/fvets.2021.762326)
Supplement: Supplementary file 2 [file Table_2.DOC]

**Supplementary Table 2** Information of PRV isolates obtained in this study and reference strains

| Strain | Year of isolation | Region of isolation | GenBank accession | Genotype (Note) |
| --- | --- | --- | --- | --- |
| Bartha | - | Hungary | JF797217 (complete genome) | Genotype I |
| Becker | - | USA | JF797219 (complete genome) | Genotype I |
| Kaplan | - | Hungary | JF797218 (complete genome) | Genotype I |
| Kolchis | 2010 | Greece | KT983811 (complete genome) | Genotype I |
| Ea | 1993 | China/Hubei | KX423960 (complete genome) | Genotype II |
| Fa | 2001 | China/Fujian | KM189913 (complete genome) | Genotype II |
| SC | 1986 | China/Sichuan | KT809429 (complete genome) | Genotype II |
| HLJ-8 | 2013 | China/Heilongjiang | KT824771 (complete genome) | Genotype II |
| TJ | 2012 | China/Tianjin | KJ789182 (complete genome) | Genotype II |
| HeN1 | 2012 | China/Henan | KP098534 (complete genome) | Genotype II |
| BJ/YT | 2012 | China/Beijing | KC981239 (complete genome) | Genotype II |
| JS-2012 | 2012 | China/Jiangsu | KP722022 (complete genome) | Genotype II |
| ZJ01 | 2012 | China/Zhejiang | KM061380 (complete genome) | Genotype II |
| HuB17 | 2020 | China/Hubei | MT949537 (complete genome) | Genotype II |
| hSD-1 | 2019 | China | MT468550 (complete genome) | Genotype II |
| HeNLH | 2017 | China/Henan | MT775883 (complete genome) | Genotype II |
| DL14/08 | 2014 | China | KU360259 (complete genome) | Genotype II |
| HuN-HH/2020 | 2020 | China/Hunan | MZ494728 (gC), MZ501780 (gE), MZ501785 (TK) | Genotype II |
| HuN-YY/2018 | 2018 | China/Hunan | MZ494729 (gC), MZ501781 (gE), MZ501786 (TK) | Genotype II |
| HuN-XT/2020 | 2020 | China/Hunan | MZ494730 (gC), MZ501782 (gE), MZ501787 (TK) | Genotype II |
| HuN-LD/2019 | 2019 | China/Hunan | MZ494731(gC), MZ501783 (gE), MZ501788 (TK) | Genotype II |
| HuN-XX/2020 | 2020 | China/Hunan | MZ494732 (gC), MZ501784 (gE), MZ501789 (TK) | Genotype II |
