# Supplementary material for: Serological Investigation and Genetic Characteristics of Pseudorabies Virus in Hunan Province of China From 2016 to 2020
Source: Front Vet Sci. 2021 Dec 16;8:762326. doi: 10.3389/fvets.2021.762326 (PMC8716618; doi:10.3389/fvets.2021.762326)
Supplement: Supplementary file 3 [file Table_3.doc]

| **PRV strains** | **Nucleotide** | | | **Amino acid** | | |
| --- | --- | --- | --- | --- | --- | --- |
|  | ***gC*** | ***gE*** | ***TK*** | ***gC*** | ***gE*** | ***TK*** |
| PRV isolates identified in this study | 99.7~99.9% | 99.5~100.0% | 99.8~100.0% | 99.2~99.8% | 98.4~100.0% | 100.0% |
| Compared with Chinese traditional PRV strains | 99.6~100.0% | 99.5~99.9% | 98.3-99.9% | 94.4~100.0% | 98.4~99.7% | 99.7% |
| Compared with Chinese variant PRV strains | 99.7~99.9% | 99.5~100.0% | 99.5~100.0% | 99.2~99.6% | 98.4~100.0% | 99.7~100.0% |
| Compared with European and American PRV strains | 95.8~96.2% | 97.7~98.0% | 42.7~43.1% | 92.5~93.1% | 95.2~95.8% | 99.1~99.4% |

**Supplementary Table 3** Sequence identity in the *gC*, *gE*, and *TK* genes of PRV isolates identified in the present study
