# Supplementary material for: Serological Investigation and Genetic Characteristics of Pseudorabies Virus in Hunan Province of China From 2016 to 2020
Source: Front Vet Sci. 2021 Dec 16;8:762326. doi: 10.3389/fvets.2021.762326 (PMC8716618; doi:10.3389/fvets.2021.762326)
Supplement: Supplementary file 4 [file Data_Sheet_1.docx]

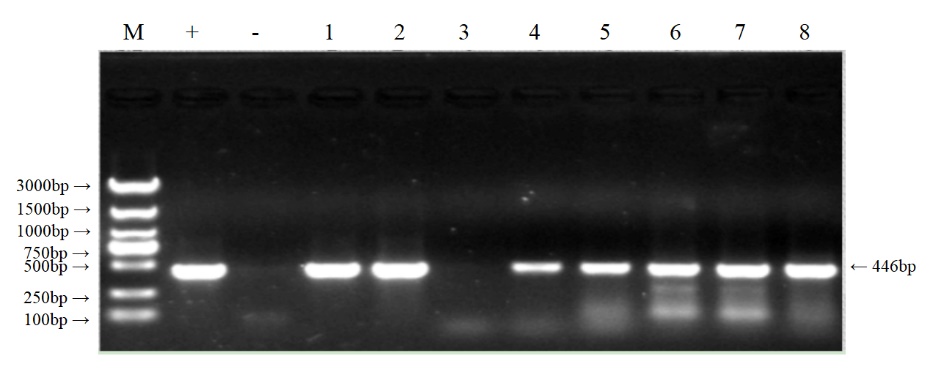


**Supplementary Figure 1**: PCR amplification products of PRV-*gG* gene

M: DL3000 DNA marker; +: Positive control; -: Negative control; 1-8: Eight representative samples.
